# Stanniocalcin-1 Co-Localizes with Insulin in the Pancreatic Islets

**DOI:** 10.5402/2012/834359

**Published:** 2012-10-16

**Authors:** Deenaz Zaidi, Jeffrey K. Turner, Michelle A. Durst, Graham F. Wagner

**Affiliations:** Department of Physiology and Pharmacology, Schulich School of Medicine and Dentistry, Western University, London, ON, Canada N6A 5C1

## Abstract

The polypeptide hormone stanniocalcin-1 (STC-1) is widely expressed in mammals and signals both locally and systemically. In many tissues STC-1 ligand is sequestered by target cell organelles (mitochondria, nuclei, and cholesterol lipid droplets) to exert diverse biological effects. Most notably, STC-1 serves as an uncoupler of oxidative phosphorylation in liver, muscle, and kidney mitochondria. The present paper describes the identification of STC-1 receptors in mouse pancreatic **β** cells and the discovery that the ligand co-localizes with insulin in pancreatic **β** cells. *In situ* hybridization (ISH) analysis subsequently revealed that pancreatic **β** cells were the source of the ligand. Intriguingly however, all ISH signal was localized over putative islet cell nuclei as opposed to the cell cytoplasm. Real-time qPCR and agarose gel electrophoresis revealed that the STC-1 amplicon generated from islet cell total RNA was the same size as that from kidney. However, relative levels of STC-1 gene expression were >100-fold lower in islets than those in kidney tissue. Collectively, these findings are indicative of a local STC-1 signalling pathway in pancreatic **β** cells. The role of STC-1 in this context remains to be established, but it could very well entail the regulation of **β** cell mitochondria membrane potential which is an integral aspect of regulated insulin release. Interestingly, STC-1 immunoreactivity was not evident in embryonic pancreatic islets, suggesting that ligand synthesis may only commence postnatally.

## 1. Introduction

The polypeptide hormone stanniocalcin-1 (STC-1) was originally identified as an endocrine regulator of serum calcium levels in fish [1]. However, the mammalian homologue appears to have more metabolically related functions. In kidney, liver, and muscle cells, STC-1 is targeted to and sequestered by the mitochondria where it uncouples oxidative phosphorylation. The resulting proton gradient energy is used instead to drive mitochondrial calcium transport and is possibly part of the mechanism by which STC-1 exerts antiapoptotic effects [2]. In luteal cells, STC-1 is targeted to and sequestered by the cholesterol lipid droplets to negatively regulate progesterone synthesis [1]. A nuclear targeting pathway becomes operative during pregnancy and lactation, whereby STC-1 is delivered systemically to mammary gland alveolar cells to promote milk fat synthesis [3]. In all the examples described above, the organelles in question possess saturable, high affinity STC-1 receptors that aid in ligand uptake and sequestration [4]. 

In the course of mapping the distribution of STC-1 receptors in mammalian tissues, we have examined the pancreas because of its well established role in intermediary metabolism. This paper describes the colocalization of STC-1 mRNA, ligand, and receptor to insulin-producing, mouse pancreatic *β* cells.

## 2. Materials and Methods

### 2.1. Histological Techniques

CD-1 male and pregnant female mice (Charles River Laboratories, Montreal, QC, Canada) were obtained for histological analysis of pancreatic tissue. Mice were anaesthetized via an i.p. injection of Somnitol (63 mg/kg) and subjected to intracardiac perfusion with phosphate buffered saline, pH 7.4, (PBS) containing 4% paraformaldehyde. Pancreatic tissue was then removed, postfixed overnight in PFA, and embedded in paraffin. Late stage mouse embryos (e17.5) were fixed and embedded in paraffin as previously described [5]. All tissue sections were cut at a thickness of 6 microns and routinely stained with haematoxylin and eosin.

#### 2.1.1. Immunocytochemistry (ICC)

 ICC was performed as previously described [5, 6] using polyclonal antisera to recombinant hSTC-1 and a mouse monoclonal antibody to rat insulin (Sigma Chemicals, St. Louis, Mo, USA). Tissue sections were incubated overnight at 4°C with 1 : 200 and 1 : 1000 dilutions of insulin and STC-1 antisera, respectively. In the case of mouse embryos, sites of antibody binding were visualized with biotinylated secondary antibodies and the Vectastain ABC peroxidase detection system (Vector Laboratories, Burlingame, CA, USA). In adult mouse pancreas, sites of antibody binding were visualized with FITC-conjugated goat anti-rabbit gamma globulin for STC-1 and Texas red-conjugated goat anti-mouse gamma globulin in the case of insulin (Vector Laboratories, Burlingame, CA, USA). As staining controls, tissue sections were incubated in normal rabbit serum (NRS) in lieu of antiserum or antiserum preabsorbed with excess antigen. Slides were washed in PBS and mounted for confocal imaging (Bio-Rad Radiance 2000 laser scanning system). 

#### 2.1.2. *In Situ* Ligand Binding (ISLB)

ISLB was performed on both adult and e17.5 embryonic mouse pancreas as previously described for the cellular localization of STC receptors [4, 7]. The method employs a fusion protein of stanniocalcin (STC) and human placental alkaline phosphatase (AP), referred to as STC-AP. Briefly, tissue sections were equilibrated in Hanks balanced salt solution containing 0.1% BSA pH 7.5 and then incubated for 90 min in the same buffer containing 1 nM STC-AP. Control slides were incubated in either AP alone or STC-AP containing 1 *μ*M hSTC. Slides were then washed, processed for visualization of bound AP activity as described in [4, 7], dehydrated, and mounted. 

#### 2.1.3. *In Situ* Hybridization (ISH)

For ISH on adult mouse pancreas, a 900 bp cDNA encoding the entire open reading frame of mouse STC-1 [8] was used as a template for digoxigenin-labelled riboprobe synthesis in sense and antisense orientations (Amersham Pharmacia Biotech, Canada). The ISH procedure was then conducted as previously described [5, 6, 8]. Three animals were analyzed and images were captured via brightfield microscopy using a digital camera.

### 2.2. Tissue RNA Isolation and Quantitative PCR

Samples of fresh rat kidney, rat liver, and isolated rat pancreatic islets were homogenized in TRIzol (Invitrogen, Carlsbad, CA, USA) with a motorized pestle and total RNA was isolated according to the manufacturer's recommendations (Invitrogen, Carlsbad, CA, USA). Pancreatic islets were isolated by the collagenase method as previously described [9]. Relative STC-1 mRNA levels were determined using TaqMan STC-1 gene expression assay and TaqMan one-step RT-PCR master mix (Applied Biosystems, Foster City, CA, USA) as previously described [10, 11]. The expression of STC-1 was computed relative to the expression of a reference gene, glyceraldehyde 3-phosphate dehydrogenase (GAPDH), using the comparative cycle threshold method (delta-delta-C_T_). Reactions were 15 ul in volume and contained 25 ng of total RNA with a 260/280 absorbance ratio of no less than 1.8. Each reaction was performed in quadruplicate. Reverse transcription was first carried out for 30 minutes at 48°C followed by an enzyme activation phase of 10 minutes at 95°C. The subsequent amplification reaction was run for 40 cycles alternating between 95°C and 60°C for 15 sec and 1 minute, respectively. All steps were performed on an ABI Prism 7900 HT sequence detector. Sequence Detection Software 2.0 (Applied Biosystems, Foster City, CA, USA) was used for analysis. Efficiencies above 90% were deemed acceptable. Amplified cDNAs (amplicons) were also resolved on 2% agarose gels for comparison of size.

## 3. Results 

**Figure 1:**
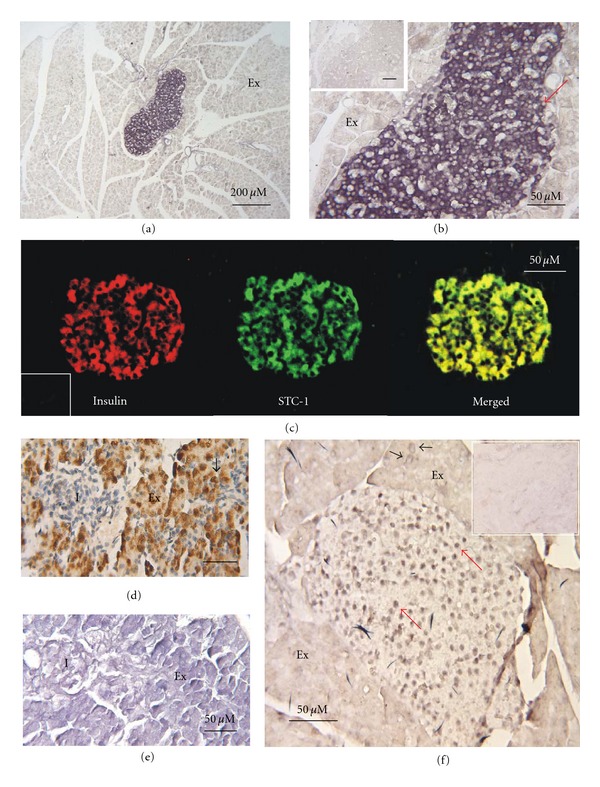
Localization of STC-1 (receptor, ligand, and mRNA) and insulin ligand in mouse pancreatic islets. (a) and (b) show an example of *in situ* ligand binding (ISLB) to localize STC-1 receptors in adult mouse pancreas ((b) is higher magnification of (a)). High binding activity is evident over islet cells (dark purple). Comparatively low binding activity is evident over exocrine pancreatic acinar cells (Ex) and islet cell nuclei (arrow in (b)). The inset in (b) is a staining control. (c) shows the results of fluorescent immunocytochemistry (ICC) to colocalize STC-1 and insulin in adult mouse pancreatic islets, insulin (red), STC-1 (green), and insulin/STC-1 colocalization (merged in yellow). There was no immunoreactivity in surrounding exocrine tissue. The inset at lower left is an STC-1 staining control. (d) shows the distribution of STC-1 in the embryonic mouse pancreas (e17.5) as revealed by peroxidase immunocytochemistry. STC-1 immunoreactivity is evident in exocrine (Ex) acinar cells (arrow) but not in islet tissue (I). (e) shows the distribution of STC-1 receptors in a tissue section adjacent to that in (d) (e17.5). Weak binding activity is equally distributed in islet (I) and exocrine cells (Ex). (f) shows the distribution of STC-1 mRNA as revealed by digoxigenin-based *in situ* hybridization (ISH) in adult mouse pancreas. The hybridization signal obtained with antisense cRNA probe was confined for the most part to presumptive islet cell nuclei (red arrows). Weak hybridization signal was also evident over the cytoplasm of isolated acinar cells (black arrows). The inset panel is an adjacent section stained by ISH using a sense cRNA probe.

 The results of *in situ* ligand binding (ISLB), immunocytochemistry (ICC), and *in situ* hybridization (ISH) are shown in panels A through F of Figure 1. The ISLB staining revealed large numbers of STC-1 receptor-positive cells within adult mouse pancreatic islets (Figure 1(a)). Binding activity was evenly distributed throughout the cell cytoplasm, whereas cell nuclei were notable by their absence of staining (red arrow in Figure 1(b)). While the majority of islet cells were receptor positive, a small population of cells both within the islets and on the islet periphery were clearly receptor-negative. In contrast, ligand binding was generally weak over acinar cells comprising the exocrine pancreas. 

**Figure 2:**
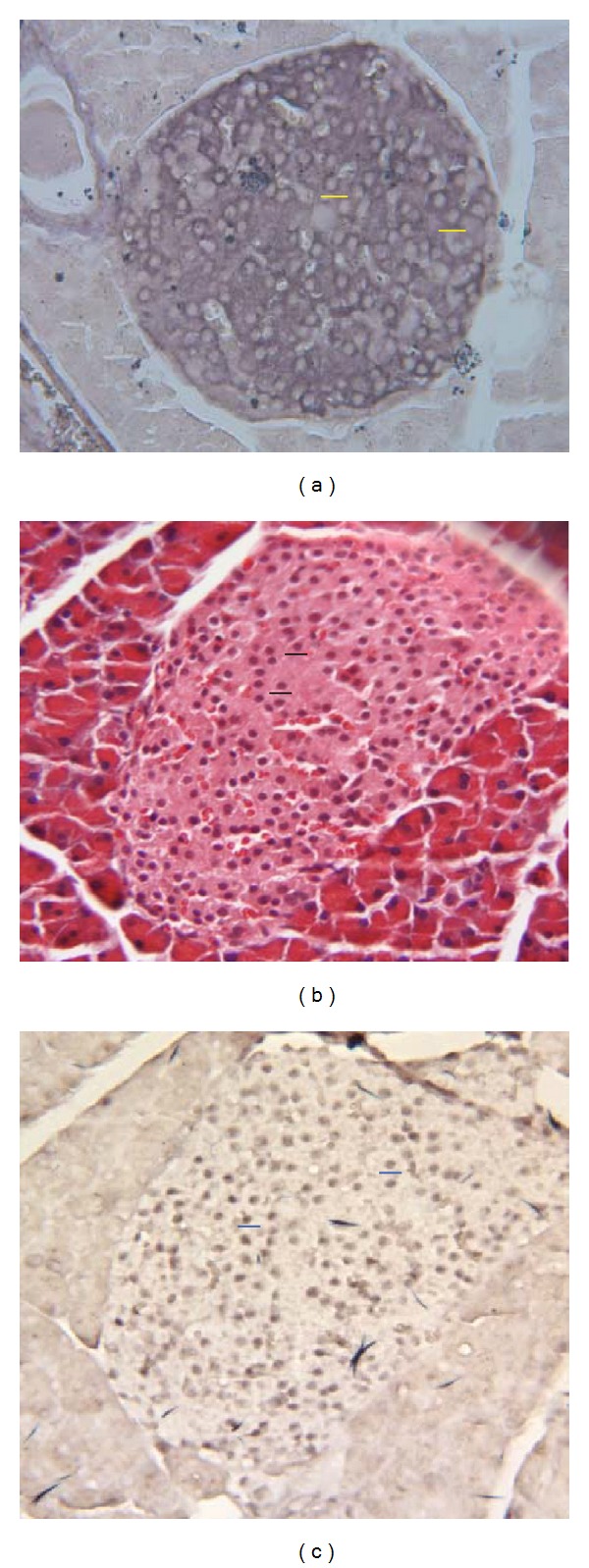
Evidence to suggest that the hybridization signal revealed by *in situ* hybridization (ISH) is over islet cell nuclei. (a) shows the distribution pattern and size (yellow bar = 10 *μ*M) of receptor-negative islet cell nuclei in adult mouse pancreas as revealed by *in situ* ligand binding. (b) shows the distribution pattern and size (blue bar = 10 *μ*M) of islet cell nuclei in adult mouse pancreas as revealed by hematoxylin-eosin staining. Manual counting revealed 262 hematoxylin-positive nuclei in this tissue section amounting to a density of 0.93 nuclei/arbitrary unit of islet area. (c) shows the distribution pattern and size (black bar = 10 *μ*M) of presumptive islet cell nuclei in adult mouse pancreas as revealed by ISH. All focal points of hybridization signal over the islets are similar in distribution pattern and size to the nuclei in (b). Manual counting revealed 285 presumptive nuclei in this tissue section amounting to a density of 0.92 nuclei/arbitrary unit of islet area.

As receptor-bearing islet cells had a distribution pattern similar to that of insulin-producing *β* cells (i.e., centrally located), correlative immunocytochemistry was carried out on adult mouse islets using antibodies to both STC-1 and insulin. The results showed that the two ligands exhibited a high degree of co-localization (Figure 2(c)), implying a close physical proximity between STC-1 and the structures associated with insulin synthesis and storage. 

In contrast to the close physical association between STC-1 and insulin in adult mice, this was not the case in pancreatic tissue from embryonic mice (e17.5). Indeed, embryonic islet cells were notable for their lack of STC-1 immunoreactivity (Figure 1(d)), whereas the acinar cell population was instead highly immunoreactive, the exact opposite of the pattern seen in adults. Similarly, in contrast to adults, ISLB staining revealed that STC-1 receptors were equally present in both endocrine and exocrine cells of e17 embryos, albeit at low levels (Figure 1(e)).

ISH staining revealed the presence of STC-1 mRNA throughout the islets, and with a distribution pattern similar to that seen by ISLB and ICC (Figure 1(f)). However, the transcript-positive sites revealed by ISH appeared to be smaller than the islet cells revealed by both ISLB and ICC, suggesting that the transcript might in fact be nuclear as opposed to cytoplasmic. To explore this further, we compared the sizes of islet cell nuclei revealed by ISLB, ISH, and hematoxylin staining (Figures 2(a)–2(c)). The results clearly showed that all transcript-positive sites (Figure 2(c)) were similar in size to cell nuclei revealed either by ISLB (Figure 2(a)) or hematoxylin staining (Figure 2(b)). This suggested that the majority of islet cell STC-1 mRNA was confined to the nucleus and not to the cytoplasm. Lastly, the number of transcript-positive nuclei was counted by hand in individual islets and compared to those revealed in adjacent tissue sections by hematoxylin staining. When expressed as numbers of nuclei per arbitrary unit of islet area, there was no significant difference in their relatives densities (0.92 ± 0.09 by ISH versus 0.91 ± 0.07 by hematoxylin (mean ± SEM; *n* = 3; *P* > 0.5, Student's *t*-test). Thus, the majority of islet cell nuclei contained STC-1 mRNA, implying that the STC-1 gene was expressed by the majority of islet cells. 

**Figure 3:**
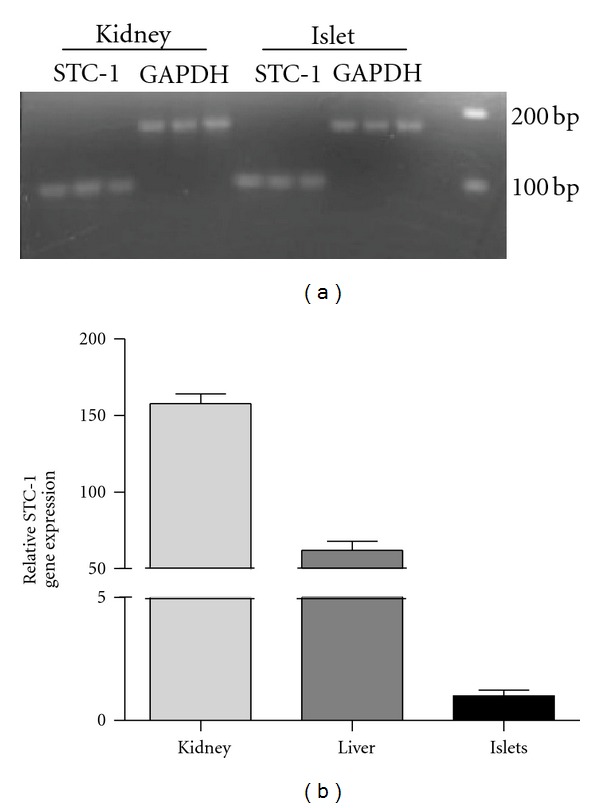
Comparison of STC-1 mRNA levels in kidney, liver, and isolated rat islets. (a) is a comparison of amplicon size following qPCR as revealed by agarose gel electrophoresis (2.0% agarose). The STC-1 and GAPDH amplicons generated from kidney and islet total RNA were similar in size. (b) illustrates the relative levels of STC-1 gene expression in rat kidney, liver, and islet total RNA as revealed by real-time qPCR.

In order to confirm that the pancreatic islets expressed the STC-1 gene, rat islets were isolated using a well established protocol and isolated RNA was subjected to real time qPCR analysis. Rat kidney and liver RNA were also analyzed for the sake of comparison. In addition, the amplicons were subjected to agarose gel electrophoresis for estimations of size. The results showed that the rat islet STC-1 amplicon was the same size as that in rat kidney (Figure 3(a)). However, the gene was expressed at much lower levels in the islets than in either liver or kidney (50–150-fold lower). Controls entailing the omission of reverse transcriptase or substituting water for RNA generated no amplicons (results not shown).

## 4. Discussion

 The present study has shown that STC-1 and insulin co-localize to a high degree in adult mouse pancreatic *β* cells. As the majority of cellular insulin is confined to secretory vesicles, most of the STC-1 immunoreactivity would appear to reside therein with insulin. This was reinforced by the ISH findings which revealed that the STC-1 gene is expressed by most if not all islet cells. Unexpectedly, the majority of STC-1 transcript was observed to be confined to cell nuclei as opposed to the cytoplasm. This is in contrast to mouse ovary [8], mouse kidney [1], and fish ovary [6], where the transcript is found for the most part in the cytoplasm. As nuclear mRNA was observed in all mice that were examined, it could be argued that the islet transcript has a slower rate of transit from the nucleus as compared to other cell types. However, in the absence of additional data such a scenario can only be viewed as speculative. Clearly therefore, further work is needed to clarify both the validity and significance of the nuclear transcript.

The tissue pattern of STC-1 mRNA distribution has been characterized by four laboratories [8, 12–14], three of which have ruled out the pancreas as a site of STC-1 gene expression [12–14]. However, as islets comprise only a small fraction of the pancreas as a whole, it is highly unlikely that the northern blotting used in these earlier studies could have revealed the low levels of gene expression observed here by qPCR in isolated islets. Interestingly, stanniocalcin-2 (STC-2), which is the product of a second related gene, also appears to be found in pancreatic tissue [15–17], although in this instance the ligand is found exclusively in glucagon-producing *α* cells [13]. Nonetheless, the cellular source of STC-2 gene expression still remains to be established.

What the stanniocalcins (STC-1 and STC-2) are doing in pancreatic islet cells is perhaps the most important question that now needs to be addressed. In this regard, it is worth noting that in some cell lines (N2a, HeLa) STC-2 localizes to the ER and Golgi and has proven to be an integral component of the unfolded-protein response; to the extent that attenuating STC-2 gene expression significantly increases cell death [18]. It is conceivable that STC-2 serves a related function in pancreatic *α* cells. STC-1, on the other hand, is known to target subcellular organelles such as cholesterol lipid droplets, cell nuclei, and mitochondria [1]. In pancreas, the STC-1 transcript, ligand, and receptor are all present in the *β* cell, which would appear to be indicative of an intracrine signalling pathway. But what it might be doing in *β* cells can only be speculated on. However, in the view of the fact that STC-1 promotes respiratory uncoupling in liver, muscle, and kidney cells through mitochondrial targeting [2] and given the critical role that respiratory uncoupling plays in insulin release [19], STC-1 could very well have regulatory effects on insulin secretion. 

Finally, the production of STC-1 by *β* cells appears to be a postnatal phenomenon. When e17.5 embryonic mouse pancreas was examined by ICC, STC-1 immunoreactivity was observed exclusively in exocrine pancreatic acinar cells. In contrast the islet cells, which at this stage of development are known to be actively synthesizing insulin and glucagon [20], were devoid of STC-1 immunoreactivity. In addition, ISLB revealed comparatively low levels of STC-1 receptors in embryonic islet cells, in marked contrast to that in adults. It would appear therefore that *β* cell synthesis of and targeting by STC-1 only becomes important postnatally. The significance of this developmental phenomenon and, more importantly, the role of STC-1 in the adult pancreas are the critical questions that now need to be addressed.
